# A Method to Reduce Non-Nominal Troposphere Error

**DOI:** 10.3390/s17081751

**Published:** 2017-07-31

**Authors:** Zhipeng Wang, Pumin Xin, Rui Li, Shujing Wang

**Affiliations:** School of Electronic Information Engineering, Beihang University, Beijing 100191, China; wangzhipeng@buaa.edu.cn (Z.W.); dearmin1992@buaa.edu.cn (P.X.); wangshujing@buaa.edu.cn (S.W.)

**Keywords:** GBAS, troposphere delay, non-nominal troposphere, VPL, satellite geometry

## Abstract

Under abnormal troposphere, Ground-Based Augmentation System (GBAS) is unable to eliminate troposphere delay, resulting in non-nominal troposphere error. This paper analyzes the troposphere meteorological data of eight International GNSS Monitoring Assessment System (iGMAS) stations and 10 International GNSS Service (IGS) stations in China and records the most serious conditions during 2015 and 2016. Simulations show that the average increase in Vertical Protection Level (VPL) of all visible satellites under non-nominal troposphere is 2.32 m and that more satellites increase the VPL. To improve GBAS integrity, this paper proposes a satellite selection method to reduce the non-nominal troposphere error. First, the number of satellites in the optimal subset is determined to be 16 based on the relationship among VPL, non-nominal troposphere error and satellite geometry. Second, the distributions of the optimal satellites are determined. Finally, optimal satellites are selected in different elevation ranges. Results show that the average VPL increase caused by non-nominal troposphere error is 1.15 m using the proposed method. Compared with the brute method and greedy method, the running rate of the proposed method is improved by 390.91% and 111.65%, respectively. In summary, the proposed method balances the satellite geometry and non-nominal troposphere error while minimizing the VPL and improving the running rate.

## 1. Introduction

Due to the influence of troposphere refraction, the propagation velocity of satellite electromagnetic signal will change and the propagation path will bend while passing through the atmosphere, resulting in approximately 2.3 m of zenith troposphere delay [1]. The pseudorange error caused by the troposphere increases integrity risk of Ground-Based Augmentation System (GBAS).

Current studies divide troposphere anomalies into horizontal and vertical components. In 2015, Jan Dousa of the Geodetic Observatory Pecny defined vertical troposphere anomalies as troposphere duct anomalies [2]. In 2011, Van Graas of Ohio State University defined horizontal troposphere anomalies as non-nominal troposphere [3]. This paper focuses on non-nominal troposphere.

In 2011, Van Graas found that severe troposphere weather conditions will induce additional troposphere delay differentials. For example, the troposphere delay differentials caused by severe troposphere weather conditions and heavy rainfall may be ±0.3 m over a 5 km baseline between the ground facility and the aircraft [3]. In 2014, the wp16 report presented at the International Civil Aviation Organization-Navigation System Panel (ICAO NSP) meeting showed unexpected atmospheric behavior that may be related to non-nominal troposphere. The combination of troposphere gradients with ionospheric gradients can significantly impact the integrity and availability of GBAS [4]. In 2016, Alizé Guilbert of the Ecole Nationale de l’Aviation Civile (ENAC) found that non-nominal troposphere error increases the Vertical Protection Level (VPL), indicating that the GBAS integrity is impacted [5].

In 2016, Daniel Gerbet of the German Aerospace Center (DLR) found that the VPL of 14 visible satellites increases by less than 5% compared with the VPL of all visible satellites, indicating that more satellites would not improve the satellite geometry [6]. Based on the current Global Positioning System (GPS) constellation and the future global BeiDou Navigation Satellite System (BDS) constellation, the average VPL increase of all visible satellites caused by non-nominal troposphere error is 2.32 m. However, when 14 satellites participate in positioning solution, the average VPL increase caused by the non-nominal troposphere error is 1.89 m. Therefore, when the satellite geometry is nearly optimal, more satellites will increase the non-nominal troposphere error in the constellation, which in turn increases VPL. VPL is related to the pseudorange error and the satellite geometry. To improve the GBAS integrity under non-nominal troposphere, a satellite selection method should be proposed to reduce the non-nominal troposphere error in positioning solution, followed by the minimization of airborne VPL [7].

**Figure 1:**
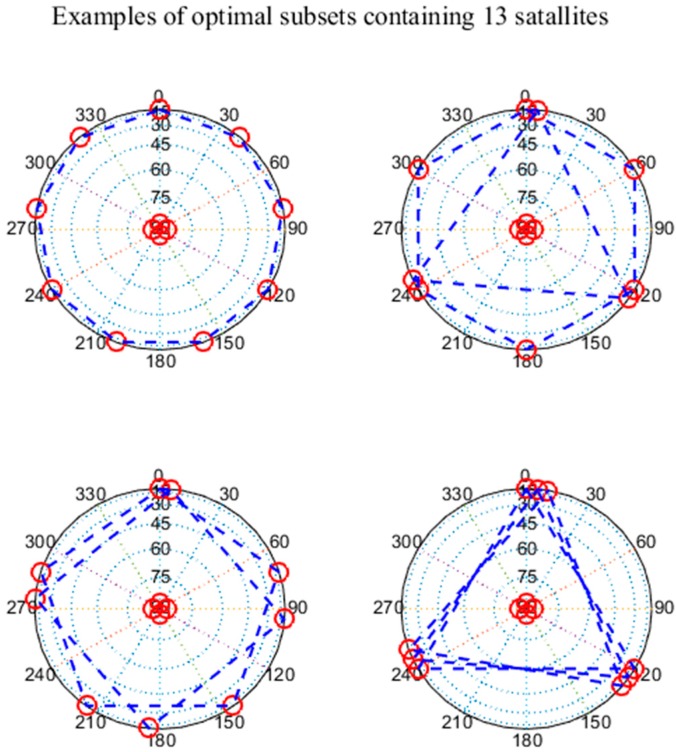
Skyplots of optimal subsets containing 13 satellites obtained with the Geometric Dilution of Precision (GDOP) method.

Numerous satellite selection methods are seemingly available, such as the elevation method, the brute method, and the Geometric Dilution of Precision (GDOP) method. The elevation method sorts the satellites by elevation angles and retains the satellites with the larger values which have relatively small pseudorange errors. However, lacking of low satellites can significantly increase the Vertical Dilution of Precision (VDOP), which in turn increases VPL and impacts the GBAS integrity. The brute method can choose the optimal satellite subset that minimizes VPL; however, this approach has a significant computational cost, which is not feasible in practical application [8]. The GDOP method chooses the final satellite subset with the minimum GDOP. In 2016, Peter F. Swaszek of the University of Rhode Island found that the satellite subset which minimizes the GDOP consists of approximately 30% low-elevation satellites and 70% high-elevation satellites. Examples of optimal subsets containing 13 satellites are shown in Figure 1 [9]. Although GDOP method can optimize the satellite geometry, the use of 70% low-elevation satellites will increase the non-nominal troposphere error in the subset, resulting in a larger VPL and thus impacting the GBAS integrity.

In summary, the elevation method cannot maintain a good satellite geometry, the GDOP method could not minimize the non-nominal troposphere error, and the brute method is infeasible to implement because of its significant computational cost. To overcome the shortcomings of these methods, this paper proposes a method to balance the psudorange error and the satellite geometry, while improving both the GBAS integrity under abnormal troposphere and the running rate.

**Figure 2:**
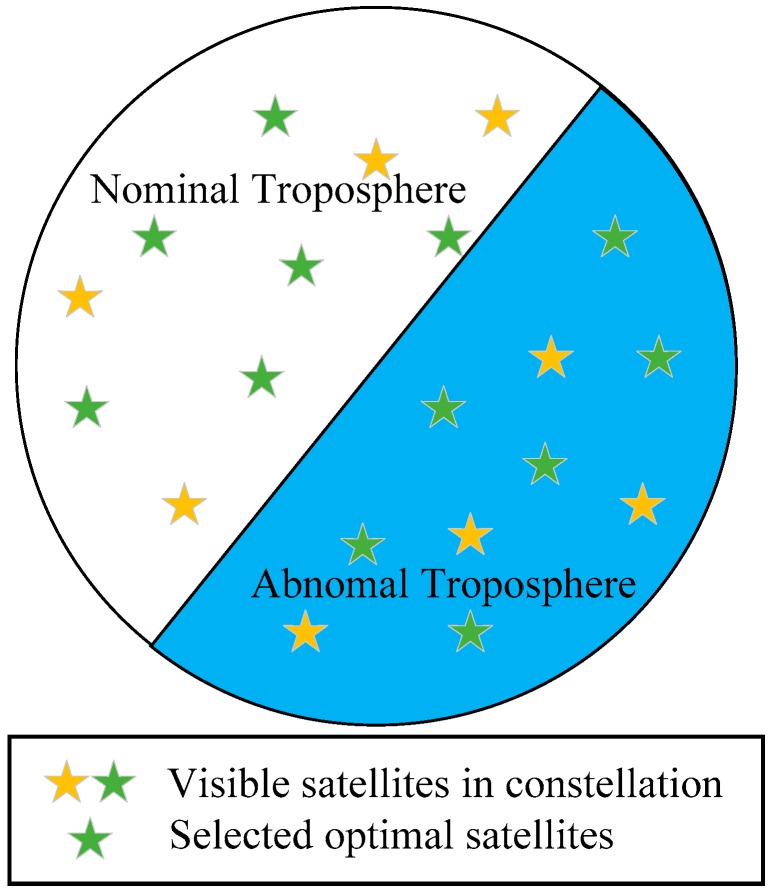
Representation of a skyplot of the selected optimal satellites under abnormal troposphere.

This paper analyzes troposphere meteorological data of eight International GNSS Monitoring Assessment System (iGMAS) stations and 10 International GNSS Service (IGS) stations in China and records the most serious conditions during 2015 and 2016. To eliminate the integrity risk caused by non-nominal troposphere error, this paper presents a method for bounding the conspiring error [10]. Given the influence of abnormal troposphere error on VPL, an effective method is proposed, which can greatly decrease the non-nominal troposphere error in positioning solution. The average VPL increase caused by non-nominal troposphere error is 1.15 m using the proposed method, which corresponds to a 1.17 m reduction. Moreover, compared with the brute method and the greedy method, the running rate of the proposed method is improved by 390.91% and 111.65%, respectively. In summary, the new method minimizes the non-nominal troposphere error in the positioning solution and airborne VPL under severe troposphere conditions, while improving both the GBAS integrity and the running rate. An illustration of the selected optimal subset is presented in Figure 2, which indicates that the final optimal satellite subset should achieve a balance between non-nominal troposphere error and satellite geometry rather than just choose the satellites with smaller non-nominal troposphere error.

The rest of the paper is organized as follows: Section 2 analyzes troposphere meteorological data in China. Section 3 briefly introduces the non-nominal troposphere model and a method for calculating the error. Section 4 presents the method for bounding the non-nominal troposphere error and analyzes its impact on VPL. Section 5 proposes a new method to select optimal satellite subset under non-nominal troposphere based on the relationship among the VPL, pseudorange error and satellite geometry. Finally, Section 6 summarizes the main points and conclusions.

## 2. Analysis of Troposphere Meteorological Data in China

This paper analyzes troposphere meteorological data of eight iGMAS stations and 10 IGS stations in China during 2015 and 2016. The meteorological data were obtained from the IGS website and iGMAS stations.

**Figure 3:**
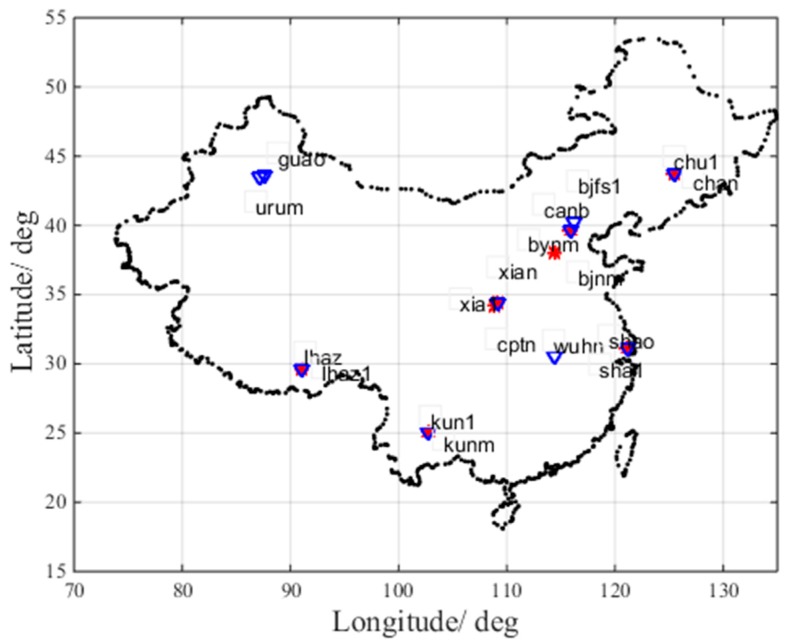
Locations of eight International GNSS Monitoring Assessment System (iGMAS) stations and 10 International GNSS Service (IGS) stations in China.

Figure 3 shows the locations of the eight iGMAS stations and 10 IGS stations in China. The red asterisks represent the iGMAS stations, while the blue triangles represent the IGS stations.

**Figure 4:**
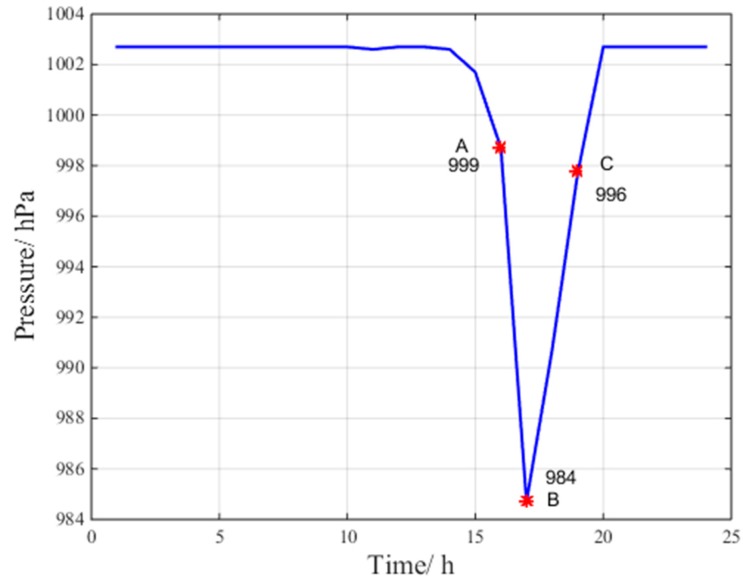
Abnormal pressure condition.

**Figure 5:**
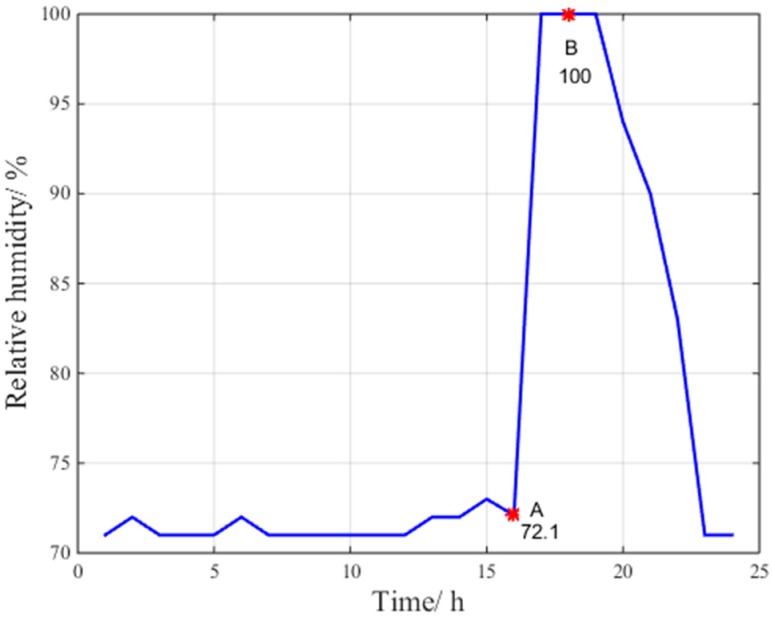
Abnormal relative humidity condition.

**Figure 6:**
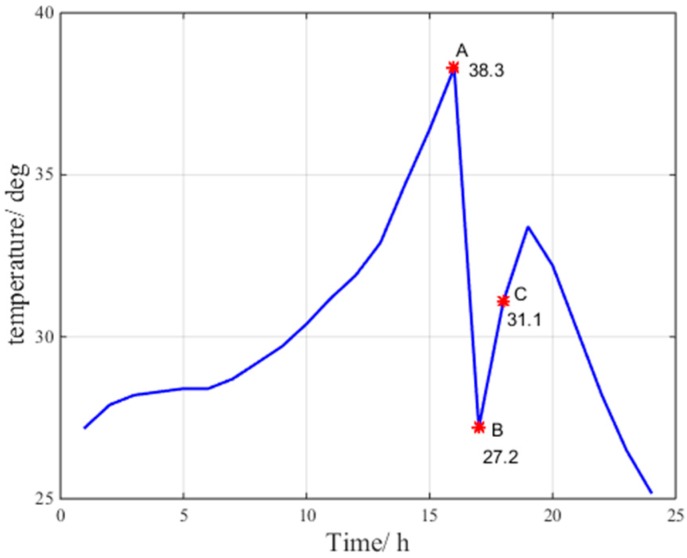
Abnormal temperature condition.

Figure 4, Figure 5 and Figure 6 indicate the abnormal troposphere conditions on 24 July 2016 at the Beijing fangshan station.

According to the data at points A, B and C for pressure, relative humidity and temperature in Figure 4, Figure 5 and Figure 6, respectively, the troposphere meteorological parameters at the Beijing fangshan station on 24 July 2016 are observed to fluctuate remarkably, indicating abnormal troposphere conditions. Clearly, the maximum change in pressure is 15 hPa/h, the maximum change in temperature is 11.1 °C/h, and the maximum change in relative humidity is 27.9%/h.

The troposphere delay is related to the temperature, pressure and relative humidity conditions at ground stations. In monitoring the GBAS integrity, it is assumed that the ground station and the aircraft experience approximately the same meteorological conditions, and, thus, the troposphere delay can be eliminated using the differential correction method. However, under abnormal troposphere conditions, the ground station cannot eliminate the troposphere delay by differential correction, and as a result, the differential correction residual troposphere error increases.

According to troposphere meteorological data from 18 stations collected during 2015 and 2016, the temperature change rate is less than 3 °C/h, the pressure is almost constant and the relative humidity change rate is less than 4% in the absence of abnormal troposphere conditions.

Based on the troposphere meteorological data during 2015 and 2016, the most serious troposphere anomalies at eight iGMAS stations in China are shown in Table 1.

The most serious troposphere anomalies at 10 IGS stations in China during 2015 and 2016 are shown in Table 2.

Table 1 and Table 2 indicate that the eight iGMAS stations and 10 IGS stations in China all experienced troposphere anomalies during 2015 and 2016.

## 3. Non-Nominal Troposphere Model and Error Bounding Method

Under non-nominal troposphere, the troposphere delay differentials between the GBAS ground facility and the aircraft are relatively larger, i.e., the residual troposphere uncertainty in current GBAS standards cannot bound the corresponding sigma troposphere.

**Figure 7:**
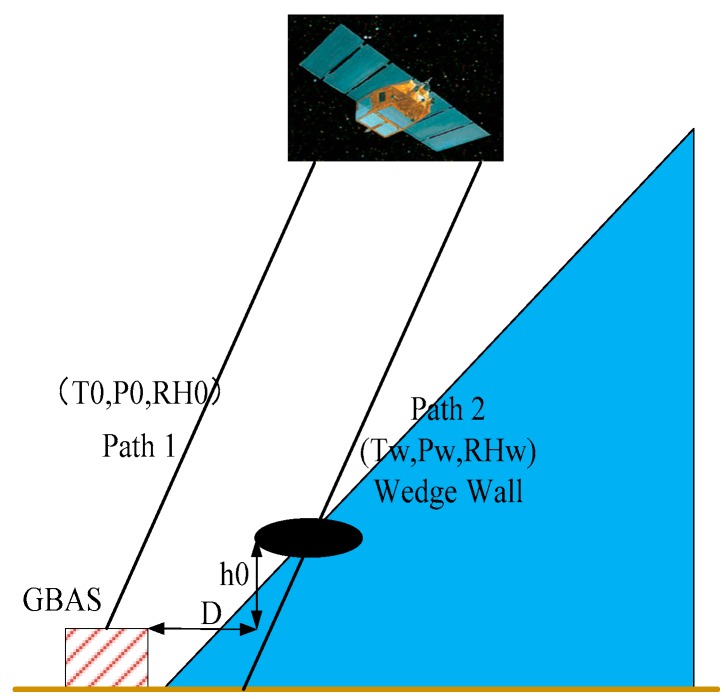
Wedge model.

**Figure 8:**
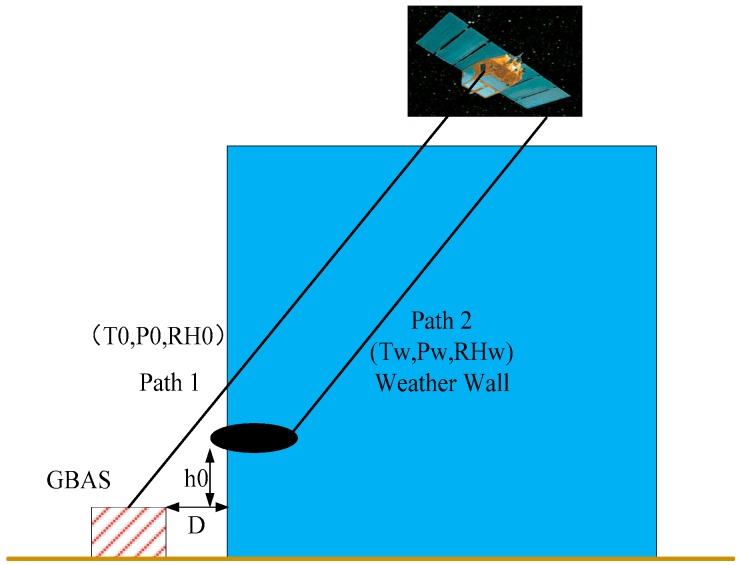
Weather wall model.

To study non-nominal troposphere, the wedge model was proposed by Thierry Gregorious of the University of Newcastle in 1998; this model considers only the effect of weather front on troposphere propagation delays, as shown in Figure 7 [11]. In 2008, researchers from Ohio State University proposed the weather wall model, which is based on the observation that local or nearby heavy rainfall is strongly correlated with the observed troposphere delay variations, as shown in Figure 8 [12]. After discussions involving an expert panel, the researchers from Ohio State University finally chose the weather wall model as the non-nominal troposphere model.

In Figure 7 and Figure 8, the parameters T, P and RH are the meteorological parameters representing temperature, pressure and relative humidity, respectively. Additionally, the parameter 0 indicates the nominal troposphere and w denotes the non-nominal troposphere.

The weather wall model represents discrete weather conditions. On the left side of the wall, the weather conditions are described by T_0_, P_0_, and RH_0_, whereas the conditions within the weather wall are given by T_w_, P_w_, and RH_w_. When the signal to the GBAS ground facility leaves the weather wall (path 1), it experiences different conditions T_0_, P_0_, RH_0_, then the signal to the user that continues in the weather wall (path 2) and experiences conditions T_w_, P_w_, RH_w_, which may introduce a larger differential residual troposphere error.

The calculation process of non-nominal troposphere error is as follows:

1. Collect the troposphere meteorological parameters (temperature, pressure and relative humidity) of the ground station for one year.
2. Calculate the maximum hourly changes in temperature, pressure and relative humidity measurements and then use these values to establish the weather wall parameters.
3. Calculate the troposphere delay differences between the GBAS ground station and the aircraft using the weather wall model and the Modified Hopfield Model (MHM).
4. The bound of the troposphere delay differences is the final non-nominal troposphere error (3, 5).
5. Divide (0°, 90°) into 90 elevation ranges at 1° intervals.
6. Assume that all satellites in each elevation range have the same maximal bias $\mu_{\max,i}$ using the absolute bias method [13].

Based on a meteorological data analysis of 18 ground stations in China, the troposphere weather parameters are as follows:

- Nominal weather conditions: T_w_ = 38 °C, P_w_ = 1024 hPa, and RH_w_ = 70%. The temperature lapse rate is set to −6.5 K/km.
- Weather wall conditions: T_0_ = 25 °C, P_0_ = 984 hPa, and RH_0_ = 100%. The temperature lapse rate is set to −6.5 K/km.

**Figure 9:**
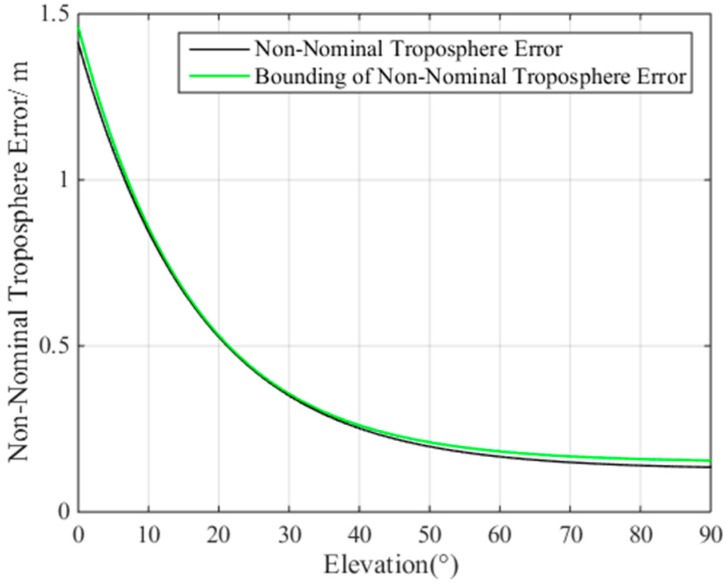
Relationship between the non-nominal troposphere error and satellite elevation.

Taking the Linzhi airport as an example, when the height between the aircraft and the ground station is 30 m, the non-nominal troposphere error can be obtained, as shown in Figure 9.

The black line in Figure 9 represents the largest non-nominal troposphere error at different elevation ranges, while the green line represents the fitted non-nominal troposphere error curve, which is a function of satellite elevation. According to the fitting results, the non-nominal troposphere error is:

(1) $$\mu_{\max,i}=1.31\times \exp(-\frac{ele_{i}}{16.21})+0.15$$

where $ele_{i}$ is the elevation angle of the *i*th satellite.

From the above analysis, it can be concluded that the non-nominal troposphere error decreases as the satellite elevation angle increases.

## 4. VPL under Non-Nominal Troposphere

In the development of the Radio Technical Commission for Aeronautics (RTCA), Federal Aviation Administration (FAA), and International Civil Aviation Organization standards, the following assumptions were made: all non-zero mean components could be mathematically removed from the differential corrections before broadcasting to the aircraft, and the pseudorange error (thermal noise, multipath, ionospheric error, troposphere error and interference) obeys a zero-mean Gaussian distribution [10]. However, it is not practical to completely remove all non-zero mean components in the pseudorange corrections because of realistic factors (i.e., GBAS site selection, ground station reference receiver antenna calibration and meteorological factors). Given these inherent difficulties, it was decided that where the removal of non-zero mean errors is not possible, the error sources must be overbounded.

VPL is an important indicator for assessing GBAS performance. This parameter provides a confidence boundary to bind the positioning error with a large probability (defined by an integrity risk of less than 2 × 10^−7^) [14].

In 2004, Frank Van Graas proposed an alternate VPL methodology to bind the conspiring error [10]. This alternate VPL-C consists of the component that arises from only the pseudorange error that obeys a zero-mean Gaussian distribution and the component that arises from conspiring biases.

### 4.1. VPL Component to Bound the Zero-Mean Pseudorange Error

The VPL component that arises from only the pseudorange error following a zero-mean Gaussian distribution is calculated as [15].

(2) $$VPL_{non}=\max(VPL_{H0},VPL_{H1})$$

where *H*0 is the fault-free condition and *H*1 is the single-receiver fault condition.

(3) $$VPL_{H0}=K_{ffmd}\sqrt{\sum_{i=1}^{N}S_{vert,i}^{2}\sigma_{i,H0}^{2}}+D_{v}$$

(4) $$VPL_{H1}=\max(\left|B_{j,vert}\right|+K_{md}\sqrt{\sum_{i=1}^{N}S_{vert,i}^{2}\sigma_{i,H1}^{2}})+D_{v}$$

(5) $$B_{j,vert}=\sum_{i=1}^{N}S_{vert,i}B_{i,j}$$

(6) $$\sigma_{i,H0}^{2}=\sigma_{gnd,i}^{2}+\sigma_{trop,i}^{2}+\sigma_{iono,i}^{2}+\sigma_{air,i}^{2}$$

(7) $$\sigma_{i,H1}^{2}=\frac{M_{i}}{U_{i}}\sigma_{gnd,i}^{2}+\sigma_{trop,i}^{2}+\sigma_{iono,i}^{2}+\sigma_{air,i}^{2}$$

where *j* is the ground subsystem reference receiver index, *i* is the satellite index, $M_{i}$ is the number of reference receivers used to compute the pseudorange corrections for the *i*th satellite, $U_{i}$ is the number of reference receivers used to compute the pseudorange corrections for the *i*th satellite, $B_{i,j}$ is the B value for the *i*th satellite and *j*th reference receiver, $K_{ffmd}$ is a multiplier determined by the probability of missed detection given that the ground subsystem is faulted, $D_{v}$ is a parameter that depends on the active approach service type, *S* is the projection matrix that relates the range domain measurements to the position domain estimates, $\sigma_{gnd,i}$ is the total (post-correction) fault-free noise term provided by the ground function for the *i*th satellite, $\sigma_{air,i}$ is the standard deviation of the aircraft contribution to the corrected pseudorange error for the *i*th satellite, $\sigma_{trop,i}$ is a term computed by the airborne equipment to cover the residual troposphere error for the *i*th satellite, and $\sigma_{iono}$ is the residual ionospheric delay uncertainty for the *i*th satellite. The calculation model of $B_{i,j}$ is as follows [16,17,18]:

(8) $$B_{i,j}=K_{B}\frac{\sigma_{gnd,i}}{\sqrt{M_{i}-1}}$$

where $K_{B}$ is a station-configurable parameter between 5 and 6; 5.6 is commonly chosen in this paper.

For GBAS Approach Service Type C (GAST C), $D_{v}$ is set to 0. For GAST D, $D_{v}$ is related to real-time approach types and ionospheric conditions, which can be calculated as [19].

(9) $$T(D_{v})=K_{fdD}\times \sqrt{\sum_{i=1}^{N}S_{vert,i}^{2}\times \sigma_{D_{R}}^{2}}$$

(10) $$\sigma_{D_{R}}=F_{pp}\times \sigma_{vig}\times 140\times v_{air}$$

where $K_{fdD}$ is equal to 5.5 according to a continuity risk of $4\times 10^{-8}$, $F_{pp}$ is the vertical-to-slant obliquity factor for a given satellite, $\sigma_{vig}$ is the vertical ionospheric gradient, and $v_{air}$ is the aircraft horizontal approach velocity, which is assumed to be 77 m/s for GBAS Approach Service Type C and 72 m/s for GAST D.

### 4.2. VPL Component to Bound the Non-Nominal Troposphere Error

The VPL component that arises from conspiring biases is obtained as [10].

(11) $$VPL_{bias}=\sum_{i=1}^{N}|S_{vert,i}\mu_{\max,i}|$$

where $\mu_{\max,i}$ is the non-nominal troposphere error of the *i*th satellite.

From the above analysis, we can see that the VPL under non-nominal troposphere is calculated as follows

(12) $$VPL_{C}=VPL_{non}+VPL_{bias}$$

where $VPL_{non}$ is the VPL component to bound the zero-mean Gaussian error and $VPL_{bias}$ is the VPL component to bound the non-nominal troposphere error.

### 4.3. Simulation Results

This section calculates the VPL under non-nominal troposphere based on the above analysis.

#### 4.3.1. Simulation Options and Parameters

Parameters in simulations are set as:

- Location: Linzhi airport (E94.335338 N29.305495, 2949 m);
- Constellations: current GPS constellation and future global BDS constellation;
- Altitude of airplane: h = 30 m;
- Time constant of the smoothing filter: 30 s;
- Aircraft velocity: 72 m/s;
- Mask angle: 5°; and
- Simulation interval: 10 s.

#### 4.3.2. Results Analysis

**Figure 10:**
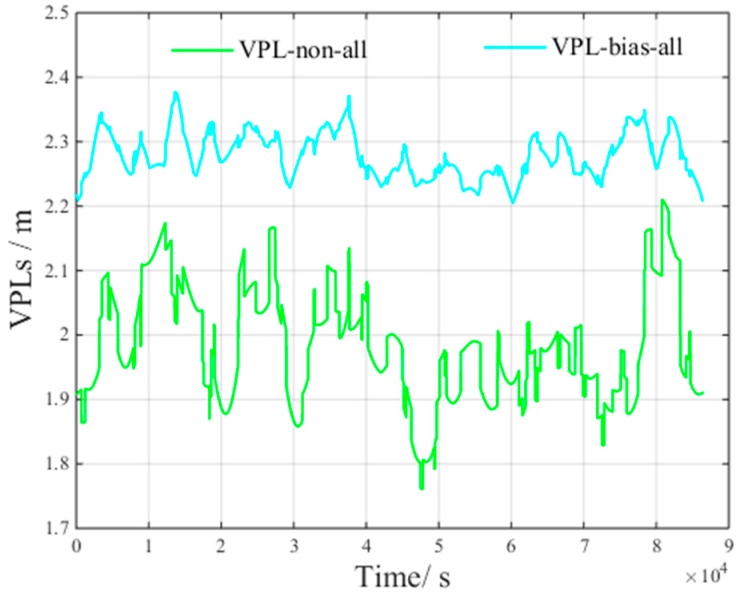
VPL of all visible satellites under non-nominal troposphere.

Figure 10 presents the VPL of all visible satellites based on the current GPS constellation and the future global BDS constellation under non-nominal troposphere. The figure presents the following results:

- $VPL_{non-all}$ is the VPL component to bound the zero-mean Gaussian error of all visible satellites.
- $VPL_{bias-all}$ is the VPL component to bound the non-nominal troposphere error of all visible satellites.

According to Figure 10, for all visible satellites, the average $VPL_{non}$ is 1.97 m and the average $VPL_{bias}$ is 2.32 m.

In 2016, Daniel Gerbeth of German Aerospace Center found that when the number of randomly selected satellites is set to 14, the average increase is between 1.5 and 12 cm and stays below 5 cm for medium latitudes. A significant influence on the GBAS availability and integrity is therefore very unlikely to occur when the number of satellites is limited to 14. In other words, the VPL of 14 randomly selected visible satellites increased by less than 5% compared with that of all visible satellites, which indicates that the selection of a subset of 14 satellites can maintain 95% of the initial accuracy and meet the GBAS integrity requirements [6].

**Figure 11:**
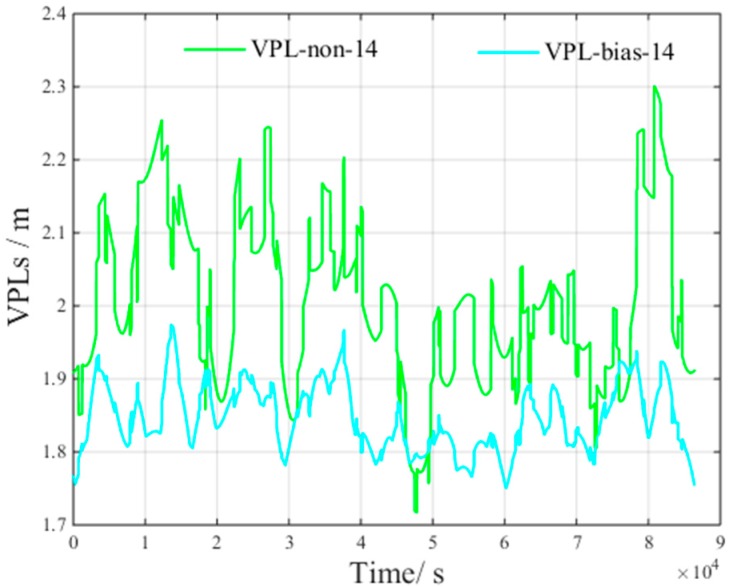
VPL of 14 visible satellites under non-nominal troposphere.

Figure 11 presents the VPL of a satellite subset containing 14 randomly selected visible satellites. The figure presents the following results:

- $VPL_{non-14}$ is the VPL component to bound the zero-mean Gaussian error of 14 visible satellites.
- $VPL_{bias-14}$ is the VPL component to bound the non-nominal troposphere error of 14 visible satellites.

Figure 11 shows that, when 14 visible satellites are included in a satellite subset, the average $VPL_{non}$ and $VPL_{bias}$ is 2.09 m and 1.89 m, respectively.

According to the results shown in Figure 10 and Figure 11, compared with the VPL of all visible satellites, the average $VPL_{non}$ increase is 0.12 m, and the average $VPL_{bias}$ decrement is 0.43 m.

**Figure 12:**
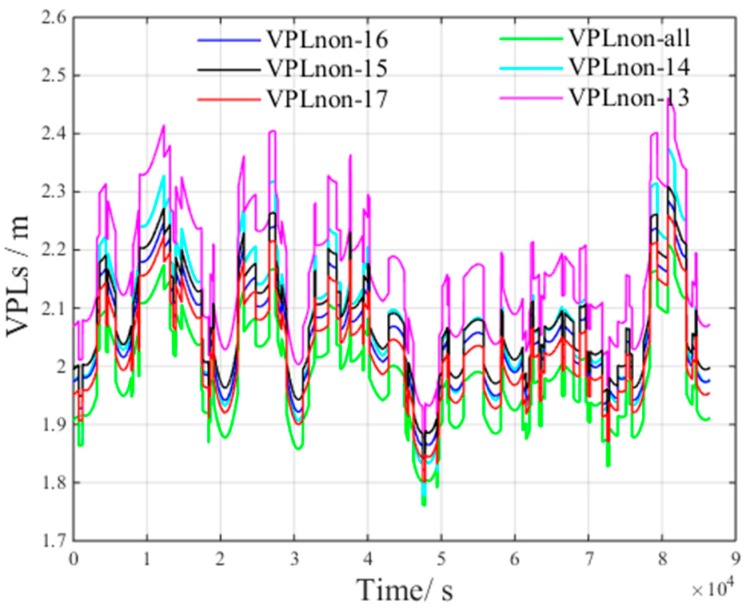
$VPL_{non}$ of different satellite subsets under non-nominal troposphere.

Figure 12 presents the $VPL_{non}$ of satellite subsets containing all, 13, 14, 15, 16 and 17 visible satellites. The 13, 14, 15, 16 and 17 satellites are randomly selected from all visible satellites. This figure presents the following results:

- $VPL_{non-13}$ is the VPL component to bound the zero-mean Gaussian error of 13 visible satellites.
- $VPL_{non-15}$ is the VPL component to bound the zero-mean Gaussian error of 15 visible satellites.
- $VPL_{non-16}$ is the VPL component to bound the zero-mean Gaussian error of 16 visible satellites.
- $VPL_{non-17}$ is the VPL component to bound the zero-mean Gaussian error of 17 visible satellites.

From Figure 12, we can derive the following inequality:

(13) $$VPL_{non\_all}<VPL_{non\_17}<VPL_{non\_16}<VPL_{non\_15}<VPL_{non\_14}<VPL_{non\_13}$$

From Inequality (13), we can conclude that more visible satellites improves satellite geometry, which, in turn, increases $VPL_{non}$.

**Figure 13:**
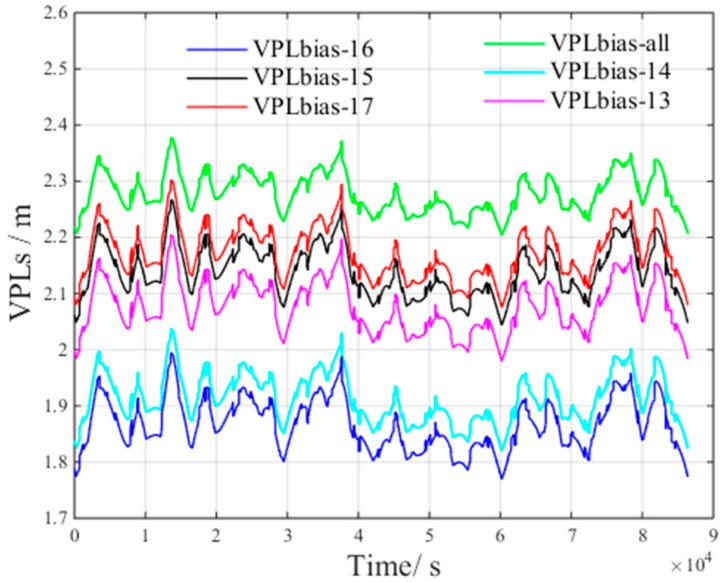
$VPL_{bias}$ of different satellite subsets under non-nominal troposphere.

Figure 13 presents the $VPL_{bias}$ of satellite subsets containing all, 13, 14, 15, 16 and 17 visible satellites. The satellites are also randomly selected from all visible satellites. This figure presents the following results:

- $VPL_{bias-13}$ is the VPL component to bind the non-nominal troposphere error of 13 visible satellites.
- $VPL_{bias-15}$ is the VPL component to bind the non-nominal troposphere error of 15 visible satellites.
- $VPL_{bias-16}$ is the VPL component to bind the non-nominal troposphere error of 16 visible satellites.
- $VPL_{bias-17}$ is the VPL component to bind the non-nominal troposphere error of 17 visible satellites.

From Figure 13, we can derive the following inequality:

(14) $$VPL_{bias\_16}<VPL_{bias\_14}<VPL_{bias\_13}<VPL_{bias\_15}<VPL_{bias\_17}<VPL_{bias\_all}$$

From Inequality (14), we can conclude that although the satellite geometry can be improved by increasing the number of satellites in the selected subset, more visible satellites would induce larger non-nominal troposphere error and, thus, increases $VPL_{bias}$.

The results show that the decrement of $VPL_{non}$ decreases whereas the $VPL_{bias}$ increases when the number of visible satellites increases beyond a certain value. This behavior may result from the following reasons:

- As the number of visible satellites increases, the satellite geometry is optimized. However, including more satellites does not further improve the satellite geometry, which can explain why the decrease of $VPL_{non}$ becomes smaller.
- Once the satellite geometry is optimized, adding additional satellites increase the non-nominal troposphere error in the constellation, which in turn increases $VPL_{bias}$.

## 5. Method to Reduce the Non-Nominal Troposphere Error

Based on the above analysis, under non-nominal troposphere, once the satellite geometry is nearly optimal, the airborne VPL increases as the number of visible satellites increases. Thus, it can be inferred that satellite selection can achieve a balance between the satellite geometry and the non-nominal troposphere error. Bellow, several selection methods will be analyzed, and then a satellite selection method to reduce the non-nominal troposphere error and minimize the VPL will be proposed.

### 5.1. Current Satellite Selection Method

A poor selection algorithm can lead to poor satellite geometry or increase the non-nominal troposphere error. This section will describe several selection methods that may be suitable for non-nominal troposphere.

1. Elevation method:

The non-nominal troposphere error decreases with increasing elevation angle. The elevation method sorts the satellites by elevation angle and retains the k satellites with the larger values which have relatively smaller non-nominal troposphere errors. The aim of the elevation method is to minimize the non-nominal troposphere error in the satellite constellation. However, removing the lowest satellites can significantly increase the Vertical Dilution of Precision and in turn increase the VPL, thereby impacting the GBAS integrity [7]. Therefore, the elevation method is not an optimal method for the non-nominal troposphere. The VDOP is related to the satellite geometry of the visible satellites involved in the positioning solution. The VDOP of the *m* satellites is [20]

(15) $$VDOP=\sqrt{g_{33}}$$

(16) $$G_{m}={\left(H_{m}^{T}H_{m}\right)}^{-1}$$

where $g_{33}$ is the diagonal element of the third line and the third column of $G_{m}$,$H_{m}$ is the observation matrix of *m* satellites. Based on the computation of the VDOP, the low elevation satellites are often quite important for good vertical geometry [7].

2. GDOP method:

GDOP is an important evaluation factor for the satellite geometry, and a smaller GDOP indicates a better satellite geometry [1]. In 2016, Peter F. Swaszek of the University of Rhode Island found that the satellite subset that minimizes the GDOP consists of approximately 30% low-elevation satellites and 70% high-elevation satellites [9]. Although the GDOP method can optimize the satellite geometry and minimize the GDOP, the use of 70% low-elevation satellites will increase the non-nominal troposphere error in the constellation, resulting in a higher VPL and impacting the GBAS integrity.

3. Brute method:

The brute method examines all possible combinations of k out of N satellites to determine the best performance. The brute method can choose the optimal satellite subset which minimizes the output. In this paper, the goal is to minimize the VPL under non-nominal troposphere, and thus the criterion for satellite selection is the minimum VPL. This method is optimal in terms of returning the best possible VPL but is distinctly non-optimal in terms of its computational cost [8].

4. Greedy method:

The greedy method is similar to the brute method [21]. This method removes only one satellite at a time and uses the optimal satellite subset to evaluate the next iteration. The iteration process continues until the optimal subset of k satellites is selected. The selection criterion of the greedy method is also the minimum VPL.

### 5.2. Simulation Analysis of the Proposed Method

To improve the GBAS integrity under abnormal troposphere, this paper selects the optimal satellite subset that minimizes the airborne VPL based on the brute method for every sample epoch.

After selecting the final optimal subset, the elevation angles of the optimal satellites are calculated and the stable distribution proportions in different elevation ranges are analyzed for every sample epoch at one location.

To improve the accuracy of the statistical results and eliminate the impact of temporal factors, the distribution characteristics at all sample epochs during 2015 and 2016 are analyzed. Finally, the mean value of the distribution proportions in different elevation ranges during 2015 and 2016 are obtained.

To eliminate the influence of geographical factor and determine whether the distribution proportions of the optimal satellite subsets are applicable worldwide, this paper analyzes the distribution characteristics at different locations in detail using a 1° × 1° grid.

**Figure 14:**
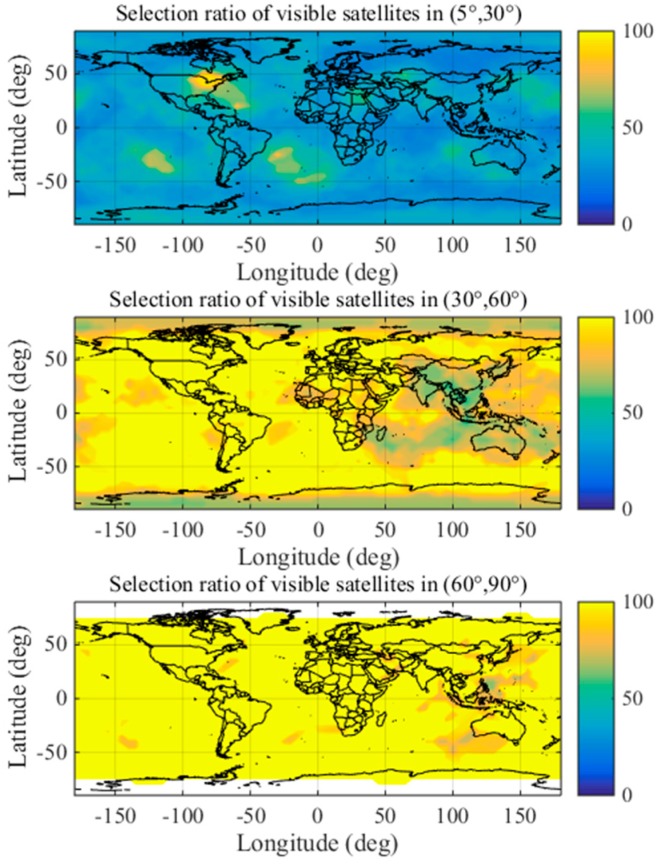
Global distribution ratios of the optimal satellite subsets with the brute method.

**Figure 15:**
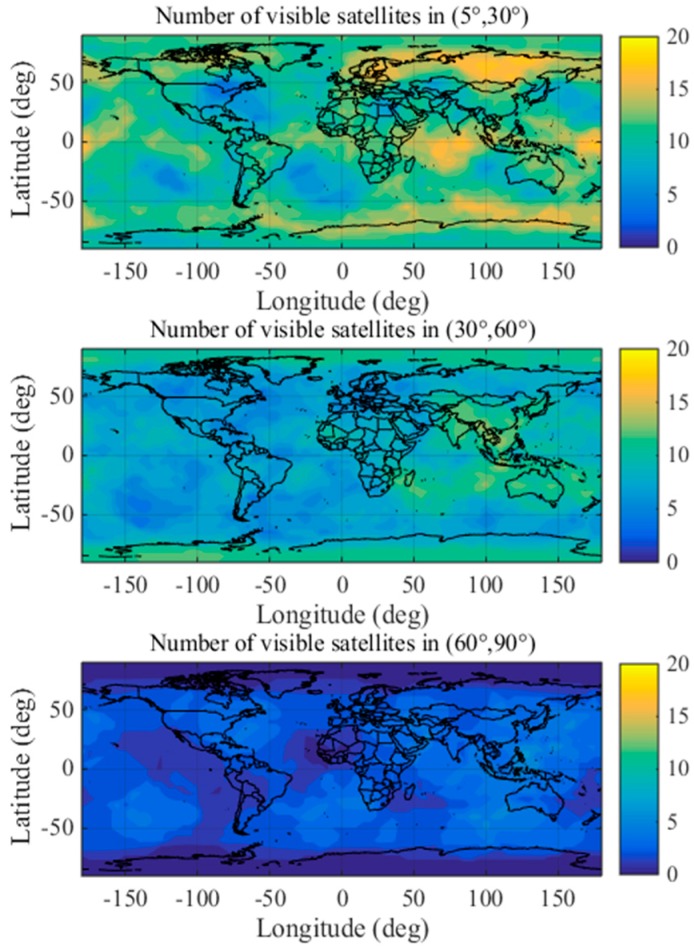
The number of visible satellites in different elevation ranges.

Thus, the distribution characteristics fully account for the temporal and geographical characteristics. The distribution characteristics of optimal satellite subsets under non-nominal troposphere are shown in detail in Figure 14 and Figure 15 using a 1° × 1° grid.

Figure 14 shows the stable distribution proportions of the satellites in the optimal subsets obtained by brute method in the different elevation ranges during 2015 and 2016 globally. The coloring indicates the distribution ratios (%) of the optimal satellites in different elevation ranges.

Figure 15 shows the number of visible satellites in different elevation ranges worldwide. The coloring indicates the number of visible satellites distributed in different elevation ranges.

The results shown in Figure 14 and Figure 15 indicate that the numbers of optimal satellite subsets are stably distributed in elevation ranges of (5°, 30°), (30°, 60°) and (60°, 90°) when the airborne VPL is minimized under the non-nominal troposphere. Based on the above analysis, the proposed method divides the satellite elevation into three categories: low elevation range (5°, 30°), mid-elevation range (30°, 60°) and high elevation range (60°, 90°).

Based on the results shown in Figure 9, the change rates of the non-nominal troposphere error in different elevation ranges are shown in Table 3.

The change rate of the non-nominal troposphere error is calculated as:

(17) $$rate=\left|\frac{error_{elev_{k+1}}-error_{elev_{k}}}{error_{elev_{k}}}\right|$$

where *i* indicates the *i*th elevation range and $elev_{k}$ is the starting elevation of the *k*th elevation range.

Figure 9 and Table 3 show that, when the satellite elevation angle is within (60°, 90°), the error change rate is the smallest. The mean value of the non-nominal troposphere error is 0.21 m, indicating that the satellites with elevation angles in (60°, 90°) can contribute to reducing the non-nominal troposphere error in the constellation. In contrast, when the satellite elevation angle is in the range of (5°, 30°), the error change rate and non-nominal troposphere error are the largest. Because the satellites in the elevation range of (5°, 30°) can help improve the satellite geometry, we must find an appropriate method for selecting the optimal satellite subset to achieve a balance between satellite geometry and the non-nominal troposphere error.

Based on the above analysis, this paper proposes the following process:

1. Obtain the optimal satellite subsets using the brute method and the greedy method.
2. Analyze the distribution characteristics of the optimal satellite subsets.
3. Determine the number *M* of satellites in the optimal satellite subset based on the greedy method and then compare the results with those obtained by the brute method to verify the accuracy of the results.
4. Assess the satellite distribution according to the optimal satellite subsets obtained by the brute and greedy methods. Next, divide the elevation ranges into (5°, 30°), (30°, 60°) and (60°, 90°). The distribution in the three elevation ranges is k1:k2:k3.
5. Select the optimal satellite subset to minimize $VPL_{C}$. For all visible satellites, assuming that there are M1, M2, and M3 satellites in each elevation range, k1 × M1, k2 × M2 and k3 × M3 satellites should be selected, respectively. The selection criterion is the GDOP contribution of each satellite.

The M and k1 : k2 : k3 values in the above process are determined by the following simulation analysis.

The GDOP is related to the distribution and number of visible satellites. The GDOP will vary when different satellite subsets are involved in the positioning solution. For convenience, the GDOP contribution of the *i*th satellite is [20]

(18) $$\Delta GDOP_{i}=trace(\frac{G_{m}h_{i}^{T}h_{i}G_{m}}{S_{ii}})$$

(19) $$S_{ii}=1-h_{i}G_{m}h_{i}^{T}$$

(20) $$h_{i}=[\begin{array}{cccc} e_{i1} & e_{i2} & e_{i3} & 1 \end{array}]$$

where $h_{i}$ is the observation vector of the *i*th satellite. A higher value of $\Delta GDOP_{i}$ indicates that the satellite geometry is improved after the addition of the *i*th satellite. Thus, the satellites with higher $\Delta GDOP_{i}$ should be prioritized.

**Figure 16:**
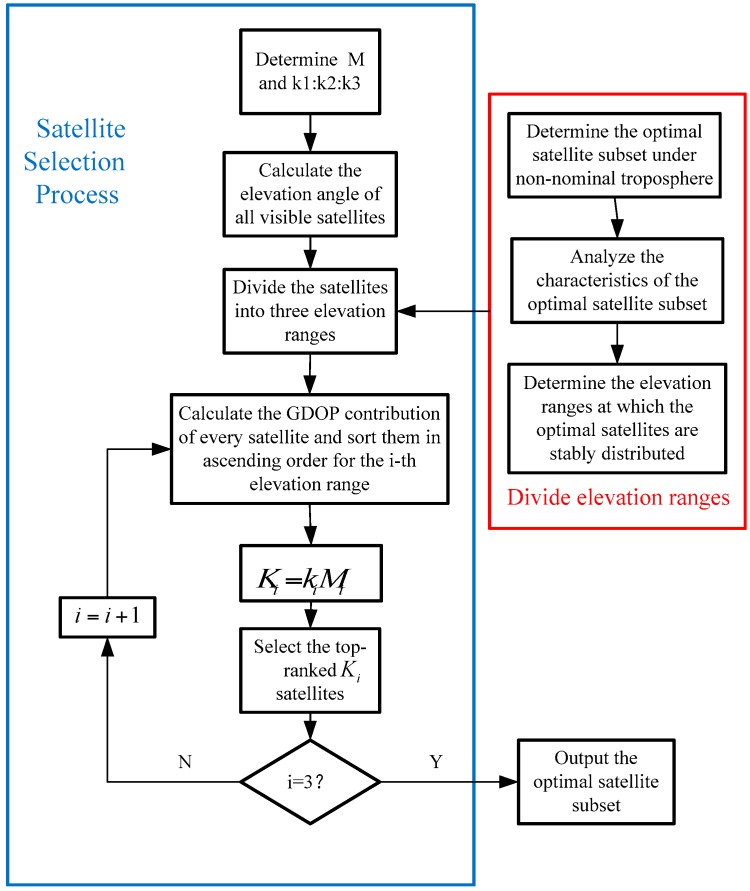
Processing flow of the proposed new method.

Figure 16 presents the processing flow corresponding to the proposed method above.

### 5.3. Simulation Results

**Figure 17:**
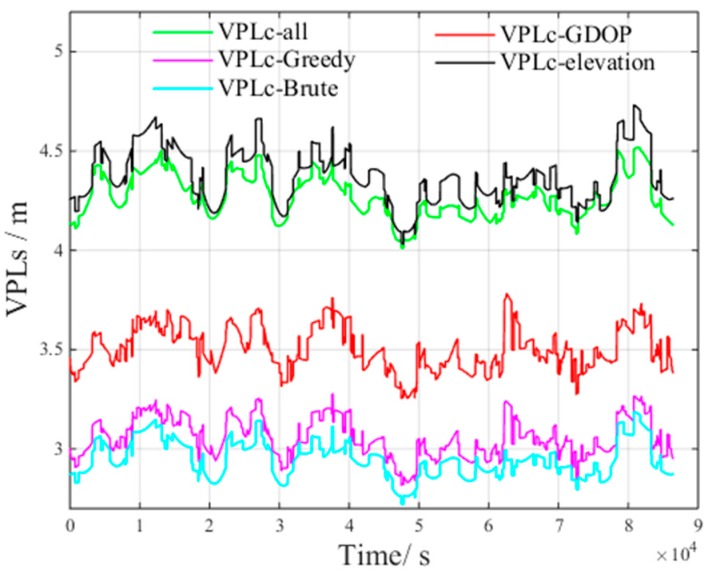
$VPL_{C}$ of different methods under non-nominal troposphere.

Figure 17 presents the $VPL_{C}$ of different methods under non-nominal troposphere. The figure presents the following results:

- $VPL_{C-all}$ is the $VPL_{C}$ of all visible satellites
- $VPL_{C-Brute}$ is the $VPL_{C}$ of the brute method.
- $VPL_{C-Greedy}$ is the $VPL_{C}$ of the greedy method.
- $VPL_{C-elevation}$ is the $VPL_{C}$ of the elevation method.
- $VPL_{C-GDOP}$ is the $VPL_{C}$ of the GDOP method.

**Figure 18:**
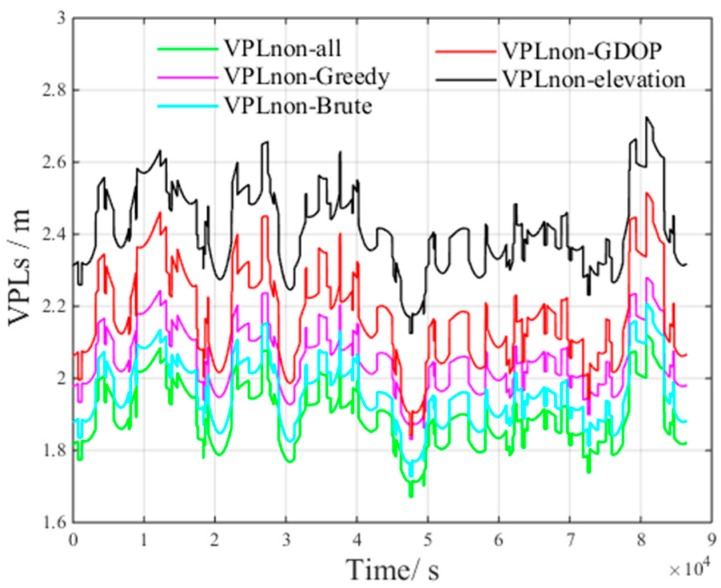
$VPL_{non}$ of different methods under non-nominal troposphere.

Figure 18 presents the $VPL_{non}$ of different methods under non-nominal troposphere. The figure presents the following results:

- $VPL_{non-all}$ is the $VPL_{non}$ of all visible satellites.
- $VPL_{non-Brute}$ is the $VPL_{non}$ of the brute method.
- $VPL_{non-Greedy}$ is the $VPL_{non}$ of the greedy method.
- $VPL_{non-elevation}$ is the $VPL_{non}$ of the elevation method.
- $VPL_{non-GDOP}$ is the $VPL_{non}$ of the GDOP method.

From Figure 17 and Figure 18, we can derive the following inequalities:

(21) $$VPL_{C\_Brute}<VPL_{C\_Greedy}<VPL_{C\_GDOP}<VPL_{C\_all}<VPL_{C\_elevation}$$

(22) $$VPL_{non\_all}<VPL_{non\_Brute}<VPL_{non\_Greedy}<VPL_{non\_GDOP}<VPL_{non\_elevation}$$

From Inequalities (21) and (22), we can infer that all visible satellites will increase the non-nominal troposphere error, which in turn increases $VPL_{C}$.

Compared with the $VPL_{non}$ of all visible satellites, both $VPL_{non\_Brute}$ and $VPL_{non\_Greedy}$ increase. However, the non-nominal troposphere errors in the selected satellite subsets decrease, and as a result, $VPL_{C\_Brute}$ and $VPL_{C\_Greedy}$ are smaller than $VPL_{C\_all}$. The above results verify the accuracy of the inference that the non-nominal troposphere error in the constellation can be reduced using an appropriate satellite selection method.

The average satellite number *M*, $VPL_{non}$ and $VPL_{bias}$ of the optimal satellite subsets obtained by different methods are summarized in Table 4.

According to Table 4, the $VPL_{non}$ of the brute method and the greedy method increase by less than 5% compared with the VPL of all visible satellites, which meets the GBAS integrity requirements [6]. Thus, the number of satellites in the optimal subset is set to 16.

Table 5 shows the average distribution of the optimal satellite subsets obtained by the brute method in different elevation ranges for the Linzhi airport during 2015 and 2016.

Table 6 presents the average distribution of the optimal satellite subsets obtained by the greedy method in different elevation ranges for the Linzhi airport during 2015 and 2016.

According to the results shown in Table 5 and Table 6, the distribution ratios of the optimal satellite subsets obtained by the brute and greedy methods in different elevation ranges are 24.6:50.3:25.1 and 25.7:49.5:24.8, respectively. For convenience, in this paper, the ratio is set to 1:2:1. Given that the number of satellites in the optimal subset is 16, it is necessary to select four, eight and four satellites in the elevation ranges of (5°, 30°), (30°, 60°) and (60°, 90°), respectively.

Based on the current GPS constellation and the global BDS constellation, the numbers of satellites in different elevation ranges are shown in Table 7 for the case in which the number of visible satellites at the Linzhi airport is minimized.

Table 7 shows that the requirements of satellite selection can be met when the number of visible satellites at the Linzhi airport is minimized.

**Figure 19:**
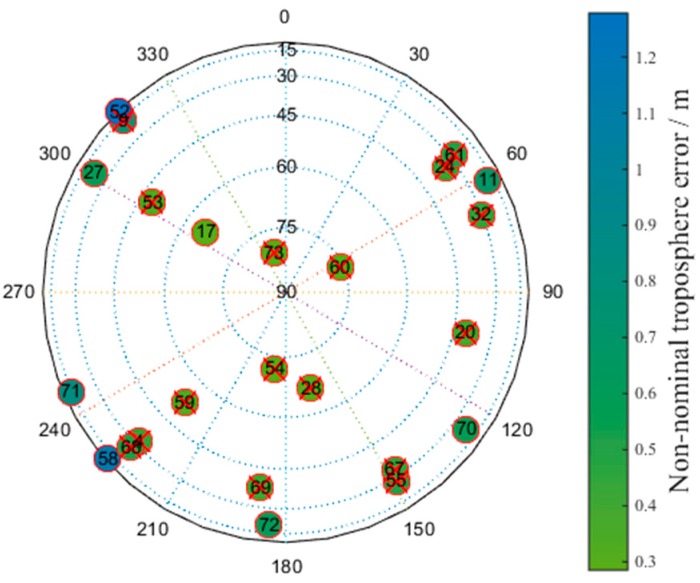
Skyplot of the optimal satellite subset obtained by the brute method.

Figure 19 presents a skyplot of the optimal satellite subset obtained by the brute method corresponding to the epoch in which the number of visible satellites at the Linzhi airport is minimized. The satellites marked with red crosses are the selected satellites. The numbers in the circles correspond to the satellites’ numbers; the values 1–40 correspond to GPS, and 40–75 correspond to BDS. The colors represent the non-nominal troposphere error for every satellite, and red crosses indicate that the corresponding satellites are selected.

**Figure 20:**
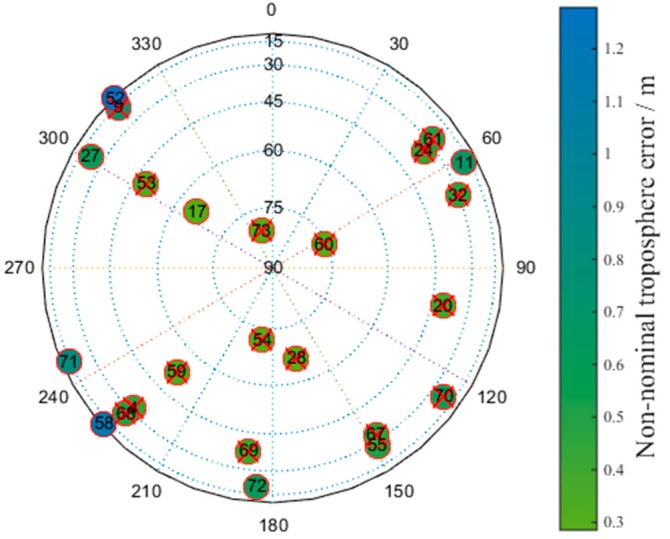
Skyplot of the optimal satellite subset obtained by the greedy method.

Figure 20 shows a skyplot for the optimal satellite subset obtained by the greedy method corresponding to the epoch in which the number of visible satellites at the Linzhi airport is minimized. The colors represent the non-nominal troposphere error for every satellite, and red crosses indicate that the corresponding satellites are selected.

Table 8 presents the satellites that were removed from the optimal satellite subset by the greedy and brute methods.

According to Table 8, the optimal satellite subset selected by the greedy method exchanges satellite No. 55 with satellite No. 70 compared with the subset obtained by the brute method. The other satellites in the optimal subsets are all the same. Satellites No. 55 and No. 70 have approximately equal non-nominal troposphere errors, confirming the effectiveness of determining the distribution of optimal satellite subsets based on the brute and greedy methods.

**Figure 21:**
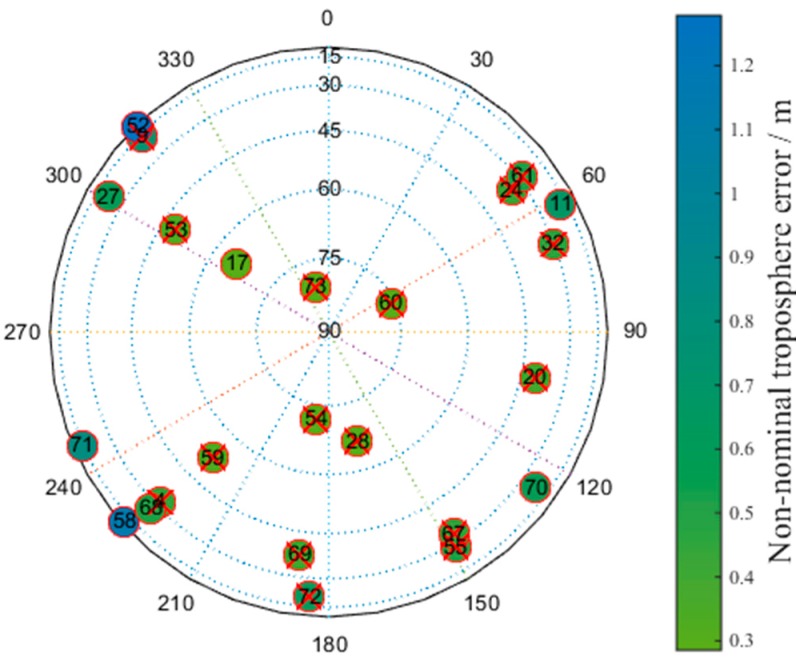
Skyplot of the optimal satellite subset obtained by the new method.

Figure 21 shows a skyplot of the optimal satellite subset obtained by the proposed method corresponding to the epoch in which the number of visible satellites at the Linzhi airport is minimized. The colors represent the non-nominal troposphere error for every satellite, and red crosses indicate that the corresponding satellites are selected.

Figure 21 shows that the optimal satellite subset selected by the new method exchanges satellite No. 68 with satellite No. 72 compared with the subset obtained by the brute method. The non-nominal troposphere error of satellite No. 68 is smaller than that of satellite No. 72. The other satellites are all the same. This slight difference indicates that the geometry of the satellite subset obtained by the new method is as good as the satellite subset obtained by the brute method.

**Figure 22:**
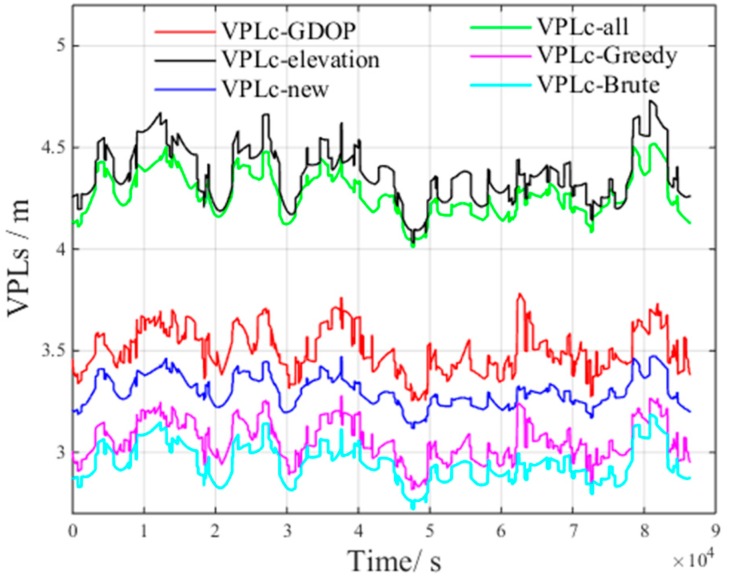
$VPL_{C}$ of the new method under non-nominal troposphere.

Figure 22 presents the $VPL_{C}$ of the new method under non-nominal troposphere, with the following results.

- $VPL_{C\_new}$ is the $VPL_{C}$ of the new method.

From Figure 22, we can derive the following inequality:

(23) $$VPL_{C\_Brute}<VPL_{C\_Greedy}<VPL_{C\_new}<VPL_{C\_GDOP}<VPL_{C\_all}<VPL_{C\_elevation}$$

By examining Inequality (23), it is clear that $VPL_{C\_new}$ decreases compared to $VPL_{C\_all}$, $VPL_{C\_elevation}$ and $VPL_{C\_GDOP}$.

**Figure 23:**
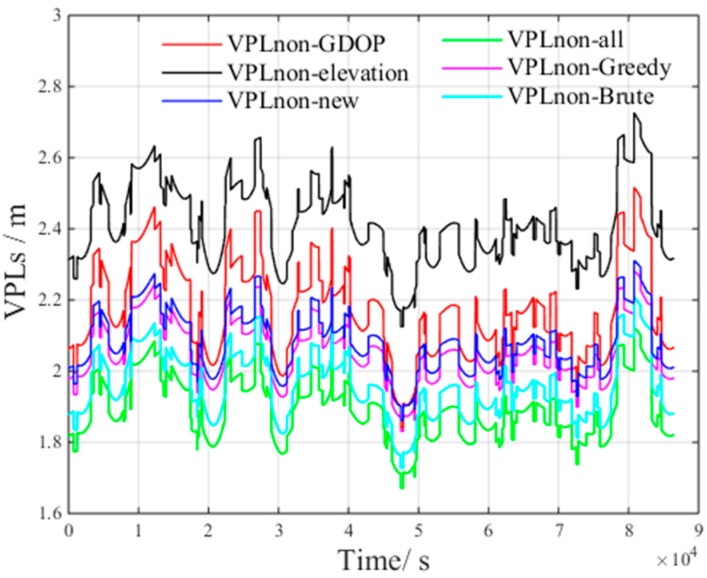
$VPL_{non}$ of the new method under non-nominal troposphere.

Figure 23 presents the $VPL_{non}$ of the new method under non-nominal troposphere, with the following results.

- $VPL_{non\_new}$ is the $VPL_{non}$ of the new method.

From Figure 23, we can derive the following inequality:

(24) $$VPL_{non\_all}<VPL_{non\_Brute}<VPL_{non\_Greedy}<VPL_{non\_new}<VPL_{non\_GDOP}<VPL_{C\_elevation}$$

By examining Inequality (24), it is clear that $VPL_{non\_new}$ decreases compared to $VPL_{non\_elevation}$ and $VPL_{non\_GDOP}$.

The average $VPL_{non}$, $VPL_{bias}$ and $VPL_{C}$ for the optimal satellite subsets obtained by the new method are shown in Table 9.

Table 9 reveals that compared to all visible satellites, with the new method the average $VPL_{bias}$ increase is 1.15 m, which corresponds to a decrease of 50.4%, and the average $VPL_{non}$ increase of the new method is 0.12 m, which corresponds to an increase of 6%. These results show that the proposed method can reduce the non-nominal troposphere error in the satellite subset. Although $VPL_{C\_new}$ increases by 0.26 m compared to $VPL_{C\_Brute}$, the running rate is greatly improved.

Professor Todd Walter of Stanford University suggested that the running rate of different methods can be characterized by the program running time (the duration from the start to the end of the program) [7].

Based on the Matlab function combntns, the program traverses all possible satellite subsets and records the running time of different methods.

Table 10 shows the running time of different methods obtained on the same day.

Table 11 presents the percentage improvement in the running rate of the new method compared to those of other methods.

As seen in Table 10 and Table 11, the running rate is greatly improved compared to those of the brute method and the greedy method.

According to the above analysis, it can be concluded that the new method proposed in this paper can achieve a balance between the satellite geometry and the non-nominal troposphere error, which greatly decreases non-nominal troposphere error in the positioning solution. The new method not only minimizes the airborne VPL under severe troposphere conditions but also improves both the GBAS integrity and running rate.

## 6. Conclusions

GBAS is unable to eliminate troposphere delay using the differential correction method when abnormal troposphere occurs, which leads to larger differential residual troposphere error.

Based on the recorded abnormal troposphere conditions, this paper analyzed the relationship between non-nominal troposphere error and satellite elevation in detail. The results show that the non-nominal troposphere error decreases as the satellite elevation increases. Therefore, to reduce the non-nominal troposphere error, the number of low-elevation satellites within the constellation should be decreased while ensuring a good satellite geometry.

To analyze the influence of non-nominal troposphere error, the VPLs of different satellite subsets are computed. Results showed that, once the constellation geometry is optimized, more satellites would increase the non-nominal troposphere error in the positioning solution and, as a results, increase the airborne VPL.

This paper also analyzed the characteristics of optimal satellite subsets. The results showed that the number of satellites in the optimal subset is 16 and that the stable distribution ratios in the elevation ranges of (5°, 30°), (30°, 60°) and (60°, 90°) can be set to 1:2:1.

In view of the above analysis, based on the proposed method, the average VPL increase caused by non-nominal troposphere error was found to be decreased by 1.17 m. Results showed that the proposed method can balance the satellite geometry and non-nominal troposphere error, which minimizes the airborne VPL and improves the GBAS integrity. In addition, the program running rate was clearly improved by 390.91% and 111.65% compared to the brute and greedy methods, respectively. These results revealed that the running rate is greatly improved, which indicates the feasibility in the practical operation.

## Figures and Tables

**Table 1:** The most serious troposphere anomalies at eight iGMAS stations in China.

Time	Station	Maximum Change in Temperature (°C/h)	Maximum Change in Pressure (hPa/h)	Maximum Change in Relative Humidity (%/h)
16 July 2015	bjf1	11.3	13.9	27.7
27 June 2015	canb	10.7	13.1	29.1
29 June 2015	chu1	12.5	15.9	31.7
28 July 2015	cptn	12.6	15.9	33.7
19 July 2015	kun1	10.7	13.4	26.8
23 July 2015	lhaz1	9.4	10.9	26.7
19 June 2015	sha1	9.2	9.6	30.1
21 June 2015	xia1	10.8	11.9	33.4
24 July 2016	bjf1	11.4	14.8	28.1
31 June 2016	canb	10.9	13.3	29.7
24 June 2016	chu1	12.3	15.7	30.9
31 July 2016	cptn	11.6	14.8	31.7
27 July 2016	kun1	10.7	12.7	29.5
30 July 2016	lhaz1	8.7	10.2	29.8
15 June 2016	sha1	9.6	10.2	32.4
17 July 2016	xia1	10.8	11.5	33.5

**Table 2:** The most serious troposphere anomalies at 10 IGS stations in China.

Time	Station	Maximum Change in Temperature (°C/h)	Maximum Change in Pressure (Pa/h)	Maximum Change in Relative Humidity (%/h)
20 July 2015	bjnm	11.3	14.1	28.7
9 July 2015	chan	10.2	13.2	28.4
16 July 2015	bjfs	11.1	13.8	27.5
19 July 2015	kunm	10.8	13.6	27.1
23 July 2015	lhaz	9.1	10.7	26.5
19 June 2015	shao1	9.7	10.3	32.8
22 July 2015	urum	10.9	13.7	31.1
17 July 2015	wuhn	11.2	14.5	29.8
21 June 2015	xian	10.5	11.5	32.8
26 July 2015	guao	11.3	13.2	27.4
23 July 2016	bjnm	11.6	14.2	29.4
16 July 2016	chan	10.7	13.8	28.7
24 July 2016	bjfs	11.1	15.0	27.9
27 July 2016	kunm	10.6	12.7	26.7
30 July 2016	lhaz	8.8	10.1	25.5
15 June 2016	shao1	9.4	9.8	32.2
30 July 2016	urum	10.5	13.5	30.2
11 July 2016	wuhn	11.7	14.9	30.4
17 June 2016	xian	10.3	11.2	32.5
17 July 2016	guao	10.6	12.7	26.3

**Table 3:** Change rates of the non-nominal troposphere error for different elevation ranges.

Elevation Range	Change Rate of Error
(5°, 30°)	74.25%
(30°, 60°)	48.76%
(60°, 90°)	14.95%

**Table 4:** The average satellite number M, $VPL_{non}$ and $VPL_{bias}$ of different satellite subsets.

Satellite Subset	M	$VPL_{non}$ (m)	$VPL_{bias}$ (m)
All visible satellites	31.6	1.97	2.32
Brute method	16.2	2.04	0.94
Greedy method	15.9	2.07	1.12
Elevation method	19.8	2.34	2.07
GDOP method	17.4	2.11	1.57

**Table 5:** Average distribution of the optimal satellite subsets under the brute method.

Elevation Range	Distribution Ratio
(5°, 30°)	24.6%
(30°, 60°)	50.3%
(60°, 90°)	25.1%

**Table 6:** Average distribution of the optimal satellite subsets under the greedy method.

Elevation Range	Distribution Ratio
(5°, 30°)	25.7%
(30°, 60°)	49.5%
(60°, 90°)	24.8%

**Table 7:** Number of satellites in different elevation ranges when the number of visible satellites at the Linzhi airport is minimized.

Elevation Range	Number of Satellites
(5°, 30°)	11
(30°, 60°)	8
(60°, 90°)	5

**Table 8:** Satellites removed from the optimal satellite subset by the greedy and brute methods.

Method	Satellite Number of Removed Satellites
All visible satellites	Non
Brute method	11,17,27,52,58,70,71,72
Greedy method	11,17,27,52,55,58,71,72

**Table 9:** Average $VPL_{non}$, $VPL_{bias}$ and $VPL_{C}$ for the optimal satellite subsets obtained by the new method.

Satellite Subset	$VPL_{non}$(m)	$VPL_{bias}$(m)	$VPL_{C}$(m)
All visible satellites	1.97	2.32	4.29
Brute method	2.04	0.94	2.98
Greedy method	2.07	1.12	3.19
Elevation method	2.34	2.07	4.41
GDOP method	2.11	1.57	3.86
New method	2.09	1.15	3.24

**Table 10:** Running time of different methods.

Method	Running Time (min)
Brute method	537.31
Greedy method	231.65
GDOP method	197.35
Elevation method	87.45
New method	109.45

**Table 11:** Percentage improvement in running rate compared to other methods.

Method	Percentage Improvement (%)
Brute method	390.91
Greedy method	111.65
GDOP method	80.3
Elevation method	-25.1
